# Changes to the national strategies, plans and guidelines for the treatment of hepatitis C in people who inject drugs between 2013 and 2016: a cross-sectional survey of 34 European countries

**DOI:** 10.1186/s12954-019-0303-9

**Published:** 2019-05-09

**Authors:** Mojca Maticic, Jerneja Videcnik Zorman, Sergeja Gregorcic, Eberhard Schatz, Jeffrey V. Lazarus

**Affiliations:** 1 Clinic for Infectious Diseases and Febrile Illnesses, University Medical Centre Ljubljana, Japljeva Str 2, 1000 Ljubljana, Slovenia; 2 Faculty of Medicine, University of Ljubljana, Ljubljana, Slovenia; 3 Correlation Network, Foundation De RegenboogGroep, Amsterdam, The Netherlands; 4 Barcelona Institute forGlobal Health (ISGlobal), Hospital Clínic, University of Barcelona, Barcelona, Spain

**Keywords:** Cross-sectional survey, Europe, Hepatitis C, People who inject drugs, Viral hepatitis policy

## Abstract

**Background:**

Hepatitis C virus (HCV) infection is the leading cause of cirrhosis, end-stage liver disease and hepatocellular carcinoma (HCC) worldwide. In Europe, people who inject drugs (PWID) represent the majority of HCV infections, but are often excluded from treatment. The aim of this study was to report on national HCV strategies, action plans and guidelines in European countries that include HCV treatment for the general population as well as for PWID. Data on access to direct-acting antivirals (DAAs) were also collected.

**Methods:**

In 2016, 38 non-governmental organisations, universities and public health institutions that work with PWID in 34 European countries were invited to complete a 16-item online survey about current national HCV treatment policies and guidelines. Data from 2016 were compared to those from 2013 for 33 European countries, and time trends are presented. Differences in the data were analysed. Data from 2016 on general access to DAAs in PWID are presented separately.

**Results:**

The response rate was 100%. Fourteen countries (42%) reported having a national HCV strategy covering HCV treatment; 12 of these addressed HCV treatment for PWID. Respondents from ten countries (29%) reported having a national HCV action plan. PWID were specifically included in seven of them. Twenty-nine countries (85%) reported having national HCV treatment guidelines. PWID were specifically included in 23 (79%) of them. Compared to 2013, respondents reported that an additional seven countries (25%) had national strategies, an additional eight countries (29%) had action plans and an additional six countries (19%) had HCV treatment guidelines. However, PWID were not included in two, four and six of those countries, respectively. DAAs were reported to be available in 91% of the study countries, with restrictions reported in 71% of them.

**Conclusion:**

Respondents reported that fewer than half of the European countries in this study had a national HCV strategy and/or action plan, with even fewer including PWID. However, when compared to 2013, the number of such countries had slightly increased. Although PWID are often addressed in clinical guidelines, strategic action is needed to increase access to HCV treatment for this group and the situation should be regularly monitored.

**Electronic supplementary material:**

The online version of this article (10.1186/s12954-019-0303-9) contains supplementary material, which is available to authorized users.

## Background

Injecting drug use is reported to be the main route of hepatitis C virus (HCV) transmission in Europe. It accounted for 78% of all new HCV infections with a known transmission route in 2015 [1]. Among an estimated 4.5 million persons who inject drugs (PWID) in the region of WHO Europe, the total number of HCV seropositive PWID is estimated to be 2.7 million (60%) and an estimated 2 million PWID are chronically infected with HCV.

HCV seroprevalence rates vary considerably among European Union (EU) member states. In PWID, estimates range between 14% reported in the Czech Republic to 84% reported in Portugal. Five out of the 13 countries with national data reported a rate of over 50% in 2015 [2–5].

Chronic HCV infection causes progressive liver damage, and cirrhosis develops in approximately 16% of infected individuals approximately 20–30 years after infection [6]. Persons with cirrhosis are at increased risk of developing end-stage liver disease and hepatocellular carcinoma (HCC). As PWID with chronic HCV infections age, the existing HCV-related morbidity and mortality burden on health systems is likely to increase [7, 8].

The introduction of highly effective, interferon-free direct-acting antiviral (DAA) regimens has revolutionised HCV treatment [9]. However, the WHO *Global hepatitis report* estimated that worldwide, in 2015, only 20% of HCV-infected people had been diagnosed and only 7% of those diagnosed had initiated treatment [10]. Significant variation in availability and access to treatment still exists between and within European countries [10]. Treatment rates in European PWID diagnosed with HCV have been estimated to be between 10% and 30%, with wide variation reported across settings [5, 11, 12].

There are several described barriers to HCV treatment, particularly for PWID, despite several studies reporting high therapy adherence rates and low rates of reinfection [13]. Additionally, dynamic modelling suggests that HCV treatment for PWID can reduce the prevalence and incidence of chronic HCV infection [14]. Modelling studies on “treatment as prevention” have shown that achieving high DAA treatment coverage among chronically HCV-infected PWID has considerable public health value [15]. Furthermore, economic evaluations suggest that treating PWID with DAA regimens is cost-effective in high-income settings [13, 16–18].

In recent years, the guidelines of the European Association for the Study of the Liver (EASL), World Health Organization (WHO) and other expert associations have recommended that PWID be considered for HCV treatment [9, 19]. These recommendations call for PWID to be treated without delay, as untreated HCV patients have the potential to further transmit HCV. Despite these and other recommendations [20], low HCV treatment uptake levels are still observed among PWID [11, 12].

Resource capacities in European countries differ substantially, as do health priorities, and levels of access to HCV treatment [21]. In most European countries, treatment is prioritised based on fibrosis stage and/or presence of extra-hepatic manifestations of infection, such as membranoproliferative glomerulonephritis or peripheral polyneuropathy, or concomitant HIV infection. The first WHO Global Health Sector Strategy on Viral Hepatitis (published in June 2016) recommends that strategies, action plans and guidelines for HCV treatment be developed in each country according to each country’s individual epidemiology, population affected, organisation of the healthcare and community system and resource capacities, which should be aligned with existing plans [22].

In light of the apparent incongruences between these WHO recommendations and 2013 data collected from 33 European countries showing relatively few national strategies, action plans or clinical guidelines for the treatment of HCV, particularly for PWID [23], this study aimed to report changes over time. Special attention was paid to the inclusion of PWID in national strategies, action plans and clinical guidelines on HCV treatment. Access to DAAs in different European countries for the general population, and PWID in particular, was also studied.

## Methods

A 16-item, an electronic questionnaire was designed and set up at the Clinic for Infectious Diseases and Febrile Illnesses at the University Medical Centre in Ljubljana, Slovenia. This questionnaire was sent via e-mail to respondents from the same 33 European countries as in the 2013 study [23], as well as to respondents in Ukraine. Scotland was categorised separately from the rest of the UK because of the Scottish National Healthcare System’s unique approach to HCV management. The data from the UK therefore excludes all data from Scotland. Informants were drawn from a database of contacts provided by the Hepatitis C Initiative from the Correlation Network. Responses were collected from September to December 2016.

The questionnaire included questions relating to national-level HCV management and HCV treatment access. In the first part, questions on the existence of a national strategy, action plan and guidelines for treatment of HCV were provided followed by questions on whether these three options include activities regarding PWID and precise questions on the existence of separate HCV treatment guidelines for PWID, applicable to those on opioid substitution therapy (OST) and active injectors. In the second part, questions on the availability of DAAs and official policy restrictions for their use were given. “Yes” or “no” were the only possible responses to these questions. Respondents were asked to provide references for national guidelines on HCV treatment and had the option of adding comments to clarify their answers. The survey also requested the names, organisational affiliations and countries of respondents. The questionnaire was administered in English, as all respondents had previously reported fluency in the English language.

This study used the same definition of PWID used in the 2013 baseline study [23]: PWID were either people who had been “actively” injecting drugs, referring to those who had used drugs in the past 6 months, or people who were former injectors. Those who were still active non-injection drug users and/or were on OST were considered to be former injectors. This definition was taken from recommendations published by the International Network on Hepatitis in Substance Users (INHSU) in 2013 [24].

As in the study from 2013, the definitions for the terms national strategy, action plan and clinical guidelines were not specified. The respondents were expected to understand them similarly, since they are commonly used in the national health sector. No checking regarding the proper use of these terms among the respondents was planned. However, in case of poor understanding or misunderstanding of the questionnaire, the possibility for clarification was provided by the Correlation Network, either via email or phone.

Data were collected by the Hepatitis C Initiative from the Correlation Network and then reviewed and analysed by the authors. The results from the first section of the questionnaire were compared to the results from the 2013 study. A positive trend for a particular question was defined as a respondent responding “no” to a question in 2013 and “yes” to the same question in 2016. A negative trend for a particular question was defined as a respondent answering “yes” to a question in 2013 and “no” to the same question in 2016. In the latter, the respondent was asked to recheck the response for 2016. If, after rechecking, the respondent confirmed the response for 2016 to be correct, that country would have been excluded from further analysis for that particular question.

## Results

All 34 of the invited European countries participated in the survey. In four countries, surveys were completed by two different respondents in each country (Czech Republic, Finland, Greece and Slovenia). In these countries, respondents provided congruent answers regarding their respective national strategies, action plans and guidelines. However, their answers to the second section of the questionnaire differed. When asked for clarification of the discrepancies, confirmation was received only from one Greek and one Slovenian respondent. The responses from these two latter respondents were included in the study.

Respondents had differing affiliations: most of them (27/38, 71%) represented non-governmental organizations (NGOs), six (16%) were based in university hospitals, four (10%) at public health institutions and one (3%) came from a private clinic that provided medical care to PWID. The ratio of various affiliations of the respondents was comparable to the study from 2013, representing 67%, 18%, 12%, and 3%, respectively.

### National strategy, action plan and clinical guidelines for treatment of HCV infection

**Fig. 1:**
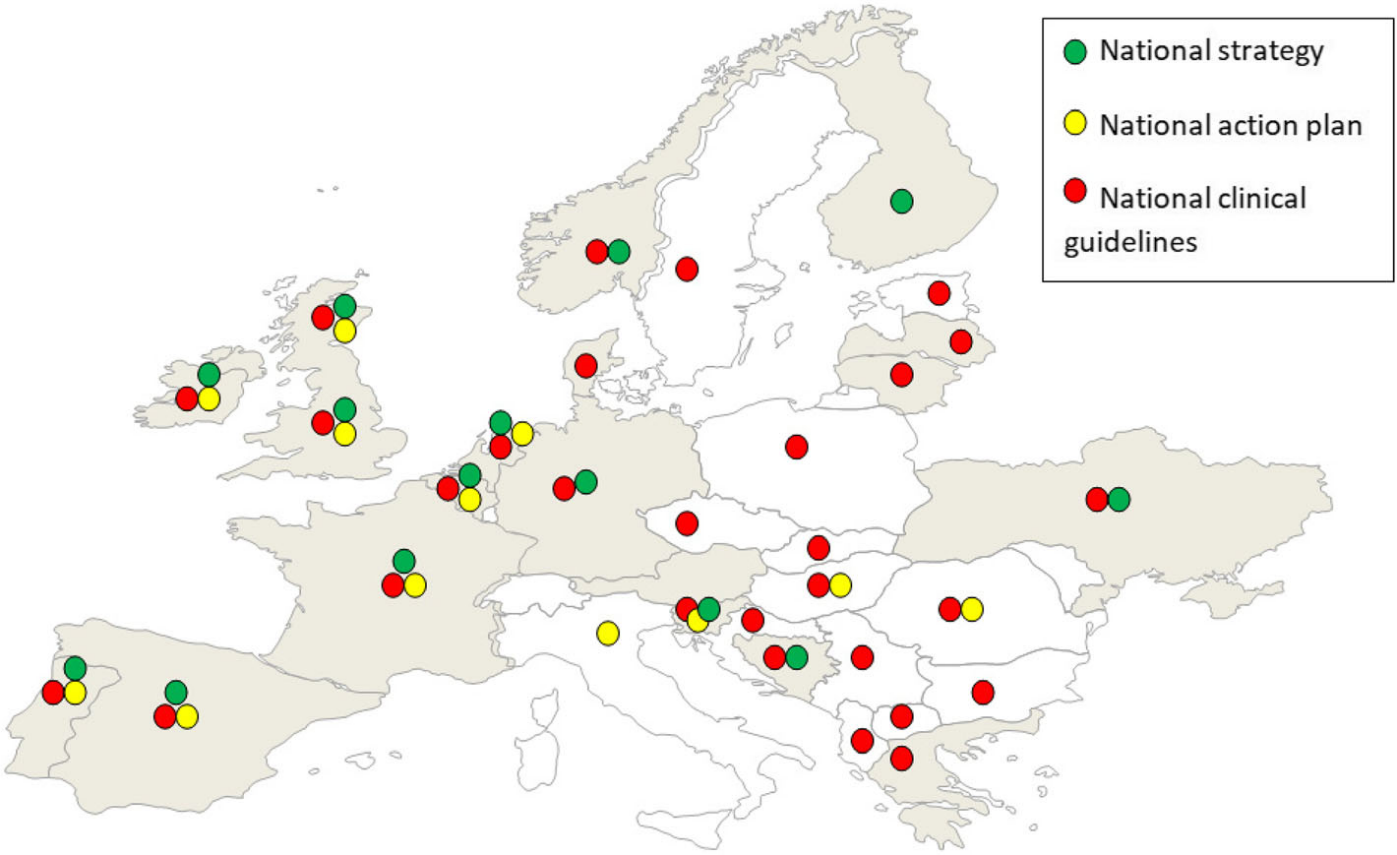
Reported presence of the national strategies, action plans and clinical guidelines for the treatment of hepatitis C from 34 European countries in 2016. ^#^Scotland was treated separately from the UK. The countries in gray participated in the study in 2013. The countries in gray and white participated in the study in 2016. ^##^The colored circles represent the existence of the national strategies (green), action plans (yellow) and clinical guidelines (red) in a particular country. ^###^People who inject drugs were not included in the national strategy in Belgium and Portugal. ^####^People who inject drugs were not included in the action plan in Belgium, Portugal and Romania. ^#####^People who inject drugs were not included in the clinical guidelines for the treatment of hepatitis C in Albania, Lithuania, Spain and Sweden

Fourteen of 34 countries (42%) reported having a national strategy for the treatment of HCV (Fig. 1). PWID were not included in only two of these countries, and one provided no answer regarding PWID. Ten countries (29%) reported having a national action plan for the treatment of patients with HCV infection (Fig. 1). PWID were included in seven of these countries.

The majority of the countries (29/34, 85%) reported having national guidelines for the treatment of HCV infection. PWID were reported to be included in all but four of these. Six countries (18%) reported that they had separate guidelines for treating PWID with HCV infection; six countries reported that there were separate guidelines for PWID concurrently being treated with OST. Two countries reported that separate treatment guidelines were applicable for PWID on OST whereas active drug users were not included in the treatment guidelines. Active drug users were reported to have separate treatment guidelines in four countries. Respondents from 17 countries provided no response to the question of treatment guidelines for active drug users (Table 1).

**Table 1:** Results of a survey from 2013 and a survey from 2016 in 33 European countries. Scotland was treated separately from the UK [25–65]

Country^#^	National strategy for HCV treatment/PWID included	National action plan for HCV treatment/PWID included	National clinical guidelines for HCV treatment	National clinical guidelines for HCV treatment: PWID included	Separate national clinical guidelines for HCV treatment in PWID	Separate national clinical guidelines for HCV treatment in PWID are applicable to OST users	Separate national guidelines for HCV treatment in PWID are applicable to active injectors
Albania							
2013 NGO	N/na	Y/N	Y	Y	N	Y	na
2016 NGO	N/na	N/na	Y	N	N	N	N
Austria							
2013 NGO	Y/Y	na/na	Y	na	na	na	na
2016 NGO	N/na	N/na	N	na	na	na	na
Belgium*							
2013 NGO [25]	N/N	na/na	N	na	na	na	na
2016 NGO	Y/N	Y/N	Y	Y	na	na	na
Bosnia and Herzegovina							
2013 university hospital [26]	Y/Y	Y/Y	Y	Y	N	na	na
2016 university hospital	Y/na	N/na	Y	Y	N	na	na
Bulgaria							
2013 public health	N/na	Y/na	Y	Y	Y	na	na
2016 NGO [27]	N/N	N/N	Y	na	na	na	na
Croatia							
2013 NGO	N/na	N/na	Y	Y	na	na	na
2016 university hospital	N/na	N/na	Y	Y	na	na	na
Czech Republic*							
2013 university hospital [28]	N/N	N/N	N	na	Y	Y	Y
2016 NGO, private clinic	N/N	N/N	Y	Y	N	N	N
Denmark							
2013 public health [29, 30]	Y/Y	Y/Y	Y	Y	Y	Y	Y
2016 public health [31]	N/na	N/na	Y	Y	N	N	N
Estonia*							
2013 NGO	N/N	N/n	N	N	N	N	N
2016 NGO	N/N	N/N	Y	Y	N	N	N
Finland*							
2013 public health [32]	N/N	N/N	N	N	N	N	N
2016 public health	Y/Y	N/na	N	na	na	na	na
France*							
2013 NGO [33]	Y/Y	Y/Y	Y	Y	N	na	na
2016 NGO	Y/Y	N/na	Y	Y	Y	Y	Y
Germany*							
2013 NGO [34, 35]	N/N	N/N	Y	Y	N	na	na
2016 NGO [36]	Y/Y	N/na	Y	Y	N	na	na
Greece							
2016 public health [37]	Y/Y	Y/Y	Y	Y	Y	Y	N
2013 NGO [37, 38]	N/N	N/N	Y	Y	N	na	na
Hungary*							
2013 NGO [39]	N/N	N/N	Y	Y	Y	Y	Y
2016 NGO [40]	N/N	Y/Y	Y	Y	na	N	N
Ireland*							
2013 NGO [41]	N/N	N/N	Y	Y	N	N	na
2016 hospital [42]	Y/Y	Y/Y	Y	Y	na	na	na
Italy*							
2013 NGO [43]	N/N	N/N	N	na	na	na	na
2016 NGO [43, 44]	N/na	Y/Y	N	na	na	na	na
Latvia							
2013 NGO [45]	Y/N	N/N	Y	Y	Y	Y	N
2016 university hospital	N/na	N/na	Y	na	na	na	na
Lithuania							
2013 university hospital [46]	Y/Y	N/N	Y	Y	Y	Y	Y
2016 university hospital	N/N	N/N	Y	N	N	N	N
Macedonia							
2013 NGO	N/na	N/na	Y	Y	Y	Y	Y
2016 NGO	N/N	N/N	Y	Y	na	na	na
Montenegro							
2013 NGO	N/N	N/N	N	na	na	na	na
2016 NGO	N/na	N/na	N	na	na	na	na
Norway							
2013 NGO	Y/Y	N/N	Y	Y	Y	na	Y
2016 NGO	Y/Y	N/na	Y	Y	na	na	na
Poland*							
2013 NGO [47]	N/N	N/N	Y	Y	N	Y	na
2016 NGO	N/N	N/N	Y	Y	Y	Y	Y
Portugal*							
2013 NGO	N/N	N/N	Y	Y	N	na	na
2016 NGO [48]	Y/N	Y/N	Y	Y	na	na	na
Romania*							
2013 NGO [49]	N/N	N/N	N	na	na	na	na
2016 NGO	N/na	Y/N	Y	Y	N	N	N
Scotland							
2013 public health [50]	Y/Y	Y/Y	Y	Y	Y	Y	Y
2016 NGO	Y/Y	Y/Y	Y	Y	Y	na	na
Serbia*							
2013 NGO	N/N	N/N	N	Y	na	na	Y
2016 NGO	N/na	N/na	Y	Y	na	na	na
Slovakia							
2013 NGO [51]	N/N	N/N	Y	Y	Y	na	na
2016 NGO	N/N	N/N	Y	Y	na	Y	N
Slovenia							
2013 university hospital [52, 53]	Y/Y	Y/Y	Y	Y	Y	Y	Y
2016 university hospital, NGO [52, 53]	Y/Y	Y/Y	Y	Y	Y	Y	Y
Spain*							
2013 NGO [54]	N/N	N/N	Y	Y	N	Y	N
2016 public health [55]	Y/Y	Y/N	Y	N	Y	Y	N
Sweden*							
2013 NGO	N/N	N/N	N	N	N	N	N
2016 NGO [56, 57]	N/N	N/N	Y	N	Y	Y	Y
Switzerland							
2013 public health	N/N	N/N	N	Y	N	Y	na
2016 NGO [58]	N/na	N/na	N	na	na	na	na
The Netherlands*							
2013 NGO [59–61]	N/Y	N/N	Y	Y	Y	Y	na
2016 NGO [62]	Y/Y	Y/Y	Y	Y	na	na	na
United Kingdom							
2013 NGO [63–65]	Y/Y	Y/Y	Y	Y	N	na	na
2016 NGO	Y/Y	Y/Y	Y	Y	na	na	na

*N* no, *Y* yes, *na* no answer, *NGO* non-governmental organization, *HCV* hepatitis C virus, *PWID* people who inject drugs, *OST* opioid substitution therapy

^#^The year of the study is presented following the country names. All respondent affiliations are indicated following the year of the study. If respondents provided references to national guidelines, the citations are given in brackets following the respondent’s affiliation

*Countries with a positive trend between 2013 and 2016 to any of the questions

### Comparison of the results from 2013 and 2016

With the exception of Ukraine, which was excluded from the comparison, all of the other 33 countries were included in both the 2013 and 2016 studies. The various affiliations of the respondents were approximately the same between the two studies.

**Fig. 2:**
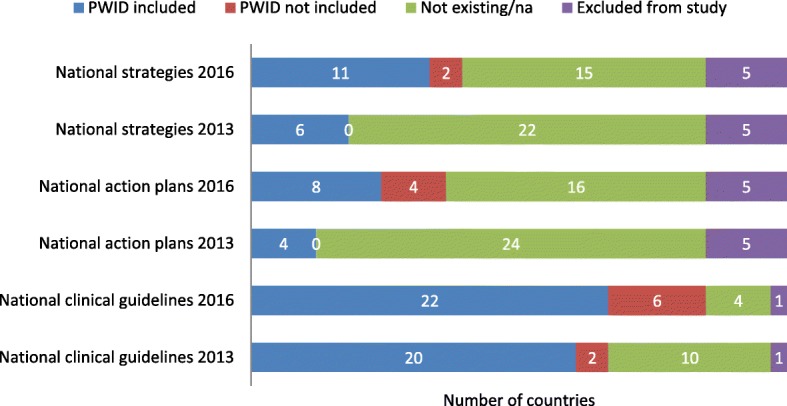
The comparison of results from 2013 and 2016 surveys on the reported presence of national strategies, action plans and clinical guidelines for the treatment of hepatitis C in 33 European countries with regard to people who inject drugs. PWID people who inject drugs

Comparing the two study years, a positive trend in a response to at least one question was observed in 16 countries (48%). A negative trend was observed in 11 responses coming from a total of eight countries; no changes in responses were detected from the remaining countries (Table 1) [25–65]. After reconfirmation of the responses for 2016, the responses with negative trends on the existence of a national strategy, action plan or clinical guideline coming from five, five and two countries, respectively, were excluded from further analysis. A positive trend in responses on the existence of a national strategy, action plan or clinical guideline was reported from seven, eight and six countries, respectively. These represent 25%, 29% and 19% increases, respectively (Fig. 2). Although positive trends were observed, PWID were still reported to be excluded from the national strategies, action plans and clinical guidelines in 2/7, 4/8 and 4/6 countries, respectively (Fig. 2).

### Availability and accessibility of direct-acting antivirals

With the exception of three responding countries, DAAs for HCV were available in the remaining countries (31/34, 91.2%) (Additional file 1).

Most countries with available DAAs reported having official policy restrictions for their use (22/31, 71%) (Additional file 1). Only four countries reported that they had no restrictions on DAA prescribing. One participant from one country did not respond.

In two countries (2/22, 9%), DAAs were used for cirrhotic patients only (fibrosis stage 4). In five countries (5/22, 23%), DAAs were prescribed only to treat patients with advanced fibrosis (stages > 3). In 12 countries (12/22, 55%), patients were retreated only if they were at the second stage of liver fibrosis or higher. Respondents from three countries (3/22, 14%) did not declare any limitations based on the stage of liver fibrosis. All, but one country, with available DAAs (31) also prescribed for various subgroups of PWID. Among the remaining 30 countries, treatment with DAAs was available for PWID on OST whereas active drug users reported being treated with DAAs in 24 countries (77%). In seven countries (23%), DAA treatment was limited only to PWID on OST. In Estonia, PWID were reported to be allowed to receive DAAs; however, they had to belong to one of the suggested PWID subgroups.

In most countries (25/31, 80%), DAAs were reported to be prescribed in accordance with official policies. This was not the case in three countries. Three additional countries did not answer this question. In 13 countries, only clinicians were reported to be able to make the final decision to treat with DAAs (13/31, 42%). In two countries (2/31, 7%), the decision to treat with DAAs was reported to be made by both clinicians and health insurance companies. In six countries (19%), the decisions were reported to be made by both clinicians and special medical commissions. In three countries (10%), the decisions were reported to be made only by special medical commissions. In four countries (13%), the decisions were reported to be made by all stakeholders: clinicians, special medical commissions and health insurance companies. In one country, the decision was reported to be made by other means apart from the three suggested options.

In 13 of the 34 countries surveyed (38%), HCV-infected PWID were reported to be treated at infectious disease clinics. In nine countries (9/34, 27 %), HCV was reported to be treated at both gastroenterology and infectious disease clinics, whereas in seven countries (7/34, 21%), HCV treatment for PWID was reported to be offered also in drug addiction centres. In two other countries (2/34, 6%), all of the previously mentioned settings, as well as general practitioners, were reported to treat PWID. In eight countries (8/34, 24%), HCV in PWID was reported to be treated also by other types of physicians. With the exception of Ukraine, all countries with available DAAs (30/31, 97%) reported that treatment for HCV is reimbursed (Additional file 1).

## Discussion

This study examined the current policies and guidelines that address hepatitis C treatment for PWID in 34 European countries. The WHO Global Health Sector Strategy on Viral Hepatitis sets out strategic directions and priority actions based on the best available scientific evidence [22]. Therefore, it serves as an essential tool for countries to use when developing more focused responses to their viral hepatitis epidemics. By comparing the 2016 survey results with those from 2013, we evaluated the influences that the WHO and EASL treatment guidelines have had on European countries in terms of their HCV management efforts [9, 66]. As such, this is the first pan-European study to evaluate the real-life dynamics of these international treatment recommendations.

Highly effective DAAs promise significant individual and public health benefits. In the context of this new treatment, the WHO Global Health Sector Strategy on Viral Hepatitis calls for the elimination of viral hepatitis as a public health threat by 2030 [22]*.* To achieve this goal, an increased capacity for HCV treatment is critical in most European countries. A modelling study that included 28 EU countries showed that to achieve the WHO targets, unrestricted treatment needs to increase from 150 000 patients in 2015 to 187 000 patients in 2025 [67]. This must be carried out in addition to scaling up screening capacities [68]. Several studies have found that there are barriers to the scale-up of HCV treatment in high-risk populations, particularly in PWID [69]. Because PWID are the major drivers of the HCV epidemic in the European region, responding to this situation with effective measures is essential for reaching the hepatitis C elimination goal set out by WHO.

This study found widespread variation in terms of HCV treatment in general and among PWID in particular. At the time of data collection, only 42% of the 34 European study countries reported having a national hepatitis strategy and 29% reported having a national action plan. When comparing these results with the 2016 HEP-Core Report, a patient-led viral hepatitis policy monitoring tool comprising of 27 European countries, a discrepancy was noted in reporting the existence of national hepatitis strategies in five countries that were included in both studies [21]. However, neither the Hep-CORE Study nor our study defines the terms national strategy or national action plan. Further, the WHO Global Health Sector Strategy does not provide a precise definition of these terms, but rather extensively present strategic directions for priority actions by countries, which most probably guided the respondents of our study.

Comparing the results obtained in 2013 and 2016, it was observed that 25% more countries reported having national strategies in 2016 than in 2013 and that 29% more countries reported having national action plans in 2016 than in 2013. Some positive trends have also been observed in recognizing PWID as a group of individuals where strategic action is needed to increase HCV treatment; however, these data were far from satisfactory with regard to the WHO Global Health Sector Strategy on Viral Hepatitis [22].

The results from our 2016 survey showed that most European countries have national guidelines for the treatment of HCV, with the treatment of PWID included in their respective guidelines. Four countries even reported having separate treatment guidelines for PWID. When comparing these data to the data from the 2016 HEP-Core Report, almost identical results were noted [21]. However, when comparing the results from 2013 to those from 2016, only a 19% increase in the number of countries with treatment guidelines was observed. The treatment guidelines of six of the28 countries with such guidelines still do not include PWID.

Despite DAAs being reported to be available in 91% of the European countries studied, restrictions on their use were reported in a majority of them. Restrictions were based on a variety of factors. The most common reason for a patient to be denied DAAs was reported to be his or her stage of liver fibrosis, and the second most common reason was concurrent injecting drug use. The Hep-CORE study also found that these were the most common reasons for European countries to restrict DAA treatment. Other reasons for DAA restriction in the Hep-CORE study included abstinence from injecting drugs for a specific period of time, alcohol use, lack of Ostia case of past or present drug use and others. According to our survey, all countries except one (97%) reported DAAs to be available for PWID on substitution therapy, but were less commonly allowed for actively injecting PWID.

Of interest, the review of the official documentation on reimbursement of HCV treatment in 35 European countries and jurisdictions was performed between 18 November 2016 and 1 August 2017 by a group of national experts [70]. It showed that 16 (46%) countries and jurisdictions required patients to have fibrosis at stage F2 or higher; 29 (83%) had no listed restrictions based on drug or alcohol use; 33 (94%) required a specialist prescriber; and 34 (97%) had no additional restrictions for people co-infected with HIV and HCV. These findings were slightly discrepant with the results of this study, as well as with the results of the Hep-CORE study [21]. In these two studies, respondents were NGO representatives, and the discrepancy points to an important gap between knowledge among a key stakeholder group (patient associations and non-governmental service providers), and what official national documents state and possibly implementation gaps. Future research may wish to directly assess the knowledge among key stakeholders of national policies and their opinions of these, including their operationalisation, rather than asking this group to report on national policies and their contents.

DAAs were reported to be prescribed predominantly in accordance with official policies. Infectious disease or gastroenterology and hepatology specialists almost exclusively prescribed DAAs within hospital settings. The implication of these findings is that even with the presence of a strategy to overcome barriers that prevent PWID to access HCV treatment, many countries may be failing to implement it [71–73]. While barriers for PWID to access HCV treatment have been reported in a number of studies [74, 75], treatment outside medical settings can be as effective as a treatment in hospitals, especially for marginalised populations such as PWID, and help overcome linkage-to-care challenges [76].

The findings of this study may provide some tangible context to the WHO Global Health Sector Strategy, which calls for health systems to deliver hepatitis treatment to different populations and settings, reinforce strategic linkages between various health services, ensure the quality of services and actively engage communities [22]. Therefore, the roles and responsibilities at every level of the health system need to be defined with respect to their delivery of hepatitis treatment—from community-based and primary health services to tertiary referral centres. There is evidence that HCV treatment can be successful in PWID and efforts to improve the generally low uptake of HCV treatment among PWID must consider the willingness of health systems and individual health providers to administer HCV treatment to members of this population [11, 77–79].

The most important limitation of this study is the involvement of only stakeholders selected from the Correlation Network’s Hepatitis C Initiative database. Most were representatives of NGOs but were not necessarily familiar with their respective government’s HCV policy. The validity of the answers provided was not cross-referenced with current, official policy, so there may be inaccuracies in respondents’ answers. However, by comparing our results to those of the Hep-CORE study, we emphasise the importance of combining data collected from various stakeholders. Further research should explore the perspectives of various civil society stakeholders, experts and government officials in Europe on current policy and practice.

## Conclusions

This is the first study to present trends in the development of national action plans, strategies and guidelines on HCV treatment for PWID in Europe. Between 2013 and 2016, there was a positive trend in recognising PWID as a group of individuals for whom strategic action is needed to increase access to and availability of HCV treatment. In the majority of European countries, DAAs were reported to be available; however, restrictions on their use were reported in almost all of them, of which the most common were fibrosis stage, and current and/or previous injecting drug use.

In order to reduce the HCV-related disease burden among PWID, a radical change in the HCV response is needed in many of the European countries investigated in this study. National strategies, action plans and guidelines that specifically address recommendations for treating PWID with HCV need to be further developed and adopted. Involving all stakeholders, including relevant NGOs, in the monitoring and reporting of national responses would be a significant step forward towards the elimination of HCV as a public health threat, as set out in the WHO Global Health Sector Strategy on Hepatitis, 2016–2021.

## Additional file

Additional file 1: Study results on access to hepatitis C treatment with direct-acting antivirals in 2016 from 34 European countries. (DOCX 21 kb)

## Abbreviations

DAA: Direct-acting antiviral
EASL: European Association for the Study of the Liver
EU: European Union
HCV: Hepatitis C virus
INHSU: International Network on Hepatitis in Substance Users
NGO: Non-governmental organisation
OST: Opioid substitution therapy
PWID: People who inject drugs
WHO: World Health Organization
