# Supplementary material for: Changes to the national strategies, plans and guidelines for the treatment of hepatitis C in people who inject drugs between 2013 and 2016: a cross-sectional survey of 34 European countries
Source: Harm Reduct J. 2019 May 9;16:32. doi: 10.1186/s12954-019-0303-9 (PMC6509821; doi:10.1186/s12954-019-0303-9)
Supplement: Supplementary file 1 — Study results on access to hepatitis C treatment with direct-acting antivirals in 2016 from 34 European countries. (DOCX 21 kb) [file 12954_2019_303_MOESM1_ESM.docx]

Additional file 1. Study results on access to hepatitis C treatment with direct-acting antivirals in 2016 from 34 European countries.

| **Country^#^** | **New DAAs available** | **Official policy on access to DAAs** | | | | **Access to DAAs for PWID** | | | **DAAs used according to the official policy** | **Decision on DAA treatment made by:** | | | | **PWID are treated for HCV at:** | | | | | **DAAs**  **reimbursed** |
| --- | --- | --- | --- | --- | --- | --- | --- | --- | --- | --- | --- | --- | --- | --- | --- | --- | --- | --- | --- |
|  |  | **Restrictions present** | **Only for F 4** | **Only for F˃2** | **Only for F≥2** | **Allowed for PWID** | **For PWID on OST** | **For active PWID** |  | **Clinician** | **Medical commission** | **Health insurance company** | **Other** | **Gastroenterology clinics** | **infectious disease clinics** | **Centres for treatment of drug addiction** | **General practitioners** | **Other** |  |
| Albania | Y | N | N | N | N | Y | Y | N | Y | Y | Y | Y | N | N | Y | N | N | Y | Y |
| Austria | Y | Y | na | na | Y | Y | Y | Y | Y | Y | N | N | N | Y | Y | Y | N | N | Y |
| Belguim | Y | Y | na | Y | na | Y | Y | Y | N | Y | N | N | N | Y | Y | N | N | N | Y |
| Bosnia and Herzegovina | Y | N | na | na | Y | N | na | na | Y | Y | Y | Y | N | Y | Y | N | N | N | Y |
| Bulgaria *(27)* | Y | Y | na | Y | na | Y | Y | na | na | N | Y | N | N | na | na | na | N | N | Y |
| Croatia | Y | Y | na | na | Y | Y | Y | N | Y | Y | Y | N | N | N | Y | N | N | N | Y |
| Czech Republic | Y | Y | na | na | Y | Y | Y | Y | Y | N/Y | N | Y | N | Y | N/Y | Y | N | Y | Y |
| Denmark *[31]* | Y | Y | na | na | Y | Y | Y | Y | Y | Y | N | N | N | Y | Y | Y | N | N | Y |
| Estonia | Y | N | N | N | N | Y | N | N | Y | Y | Y | Y | N | N | Y | N | N | N | Y |
| Finland | Y | Y | na | na | Y | Y | Y | Y | N | Y | N/Y | N | N | Y | Y | N | N | Y | Y |
| France*[36]* | Y | N | na | na | na | Y | Y | Y | Y | Y | Y | N | N | Y | Y | Y | N | N | Y |
| Germany | Y | N | na | na | na | Y | Y | Y | Y | Y | N | N | N | Y | Y | Y | Y | N | Y |
| Greece | Y | Y | Y | na | na | Y | Y | Y | Y | Y | N | N | N | Y | N | N | N | N | Y |
| Hungary *[40]* | Y | Y | N | N | N | Y | Y | N | Y | N | Y | N | N | N | Y | N | N | N | Y |
| Ireland | Y | Y | na | na | Y | Y | Y | Y | N | N | N | N | Y | Y | Y | Y | N | Y | Y |
| Italy | Y | Y | na | Y | na | Y | Y | Y | Y | Y | N | N | N | N | Y | N | N | N | Y |
| Latvia | Y | Y | N | N | N | Y | Y | Y | Y | Y | Y | N | N | N | Y | N | N | N | Y |
| Lithuania | Y | Y | na | na | Y | Y | Y | N | Y | Y | N | N | N | N | Y | N | N | N | Y |
| Macedonia | N | na | na | na | na | na | na | na | na | na | na | na | na | Y | Y | N | N | N | na |
| Montenegro | N | na | na | na | na | na | na | na | na | na | na | na | na | Y | Y | N | N | N | N |
| Norway | Y | Y | na | na | Y | Y | Y | Y | Y | N | N | N | N | Y | Y | N | N | Y | Y |
| Poland | Y | N | na | N | N | Y | Y | Y | Y | Y | N | N | N | N | Y | N | N |  | Y |
| Portugal *[48]* | Y | Y | na | na | Y | Y | Y | Y | Y | N | Y | N | N | N | Y | N | N | N | Y |
| Romania | Y | Y | Y | na | na | Y | Y | Y | Y | Y | Y | Y | N | N | Y | N | N | N | Y |
| Scotland | Y | Y | na | na | Y | Y | Y | Y | Y | Y | Y | N | N | Y | Y | Y | N | N | Y |
| Serbia | N | na | na | na | na | na | na | na | na | na | na | na | na | na | Y | na | na | na | N |
| Slovakia | Y | Y | na | Y | na | Y | Y | N | na | na | na | na | na | Y | Y | N | N | na | Y |
| Slovenia | Y | Y | na | Y | na | Y | Y | Y | Y | Y | N | N | N | N | Y | N | N | N | Y |
| Spain | Y | Y | Y | na | Y | Y | Y | N | Y | Y | N | N | N | Y | Y | N | N | N | Y |
| Sweden | Y | N | na | na | na | Y | Y | Y |  | Y | N | N | N | N | Y | Y | N | Y | Y |
| Switzerland | Y | Y | na | na | Y | Y | Y | Y | Y | Y | N | Y | N | Y | Y | Y | N | N | Y |
| The Netherlands | Y | N | na | na | na | Y | Y | Y | Y | Y | N | N | N | Y | Y | N | N | N | Y |
| United Kingdom | Y | na | N | N | N | Y | Y | Y | Y | Y | N | N | N | Y | Y | Y | Y | Y | Y |
| Ukraine | Y | Y | N | N | N | Y | Y | Y | Y | Y | Y | N | N | N | Y | N | N | Y | N |

Y: yes; N: no; na: no answer; DAA: direct acting antivirals; PWID: people who inject drugs; OST: opioid substitution therapy.
